# Supplementary material for: The association of gender and persistent opioid use following an acute pain event: A retrospective population based study of renal colic
Source: PLoS One. 2021 Aug 26;16(8):e0256582. doi: 10.1371/journal.pone.0256582 (PMC8389463; doi:10.1371/journal.pone.0256582)
Supplement: S1 Table — (DOCX) [file pone.0256582.s002.docx]

)
