# Supplementary material for: The association of gender and persistent opioid use following an acute pain event: A retrospective population based study of renal colic
Source: PLoS One. 2021 Aug 26;16(8):e0256582. doi: 10.1371/journal.pone.0256582 (PMC8389463; doi:10.1371/journal.pone.0256582)
Supplement: S2 Table — (DOCX) [file pone.0256582.s003.docx]

**S2 Table. Odds Ratios for the Association Between Characteristic and Persistent Opioid Use**

| Characteristic | Odds Ratio (95% CI) for Persistent Opioid Use ^a^ | | |
| --- | --- | --- | --- |
|  | Unadjusted | Adjusted | *P* value^b^ |
| Sociodemographic |  |  |  |
| Gender |  |  | 0.03 |
| Male (ref) | 1.00 | 1.00 |  |
| Female | 1.11 (1.05, 1.17) | 1.07 (1.01, 1.13) |  |
| Age at index renal colic |  |  | <0.001 |
| 0 - 18 | 1.03 (0.79, 1.35) | 1.07 (0.81, 1.41) |  |
| 19 – 39 (ref) | 1.00 | 1.00 |  |
| 40 - 59 | 1.08 (1.01, 1.16) | 1.00 (0.93, 1.07) |  |
| 60 - 79 | 1.37 (1.27, 1.47) | 1.14 (1.05, 1.24) |  |
| 80 + | 1.62 (1.39, 1.90) | 1.20 (1.02, 1.42) |  |
| Neighborhood income quintile |  |  | 0.03 |
| 1 – Lowest quintile (ref) | 1.00 | 1.00 |  |
| 2 | 0.87 (0.80, 0.95) | 0.91 (0.84, 0.99) |  |
| 3 | 0.88 (0.81, 0.96) | 0.94 (0.86, 1.02) |  |
| 4 | 0.83 (0.76, 0.90) | 0.89 (0.82, 0.98) |  |
| 5 - Highest quintile | 0.79 (0.73, 0.86) | 0.86 (0.79, 0.94) |  |
| Clinical History |  |  |  |
| History of stone surgery |  |  | <0.001 |
| Yes (ref) | 1.00 | 1.00 |  |
| No | 0.63 (0.56, 0.71) | 0.76 (0.68, 0.87) |  |
| Any mental health utilization |  |  | <0.001 |
| No (ref) | 1.00 | 1.00 |  |
| Yes | 1.32 (1.25, 1.39) | 1.17 (1.10, 1.24) |  |
| Substance abuse |  |  | <0.01 |
| No (ref) | 1.00 | 1.00 |  |
| Yes | 1.46 (1.29, 1.66) | 1.25 (1.09, 1.43) |  |
| Charlson Index |  |  | <0.001 |
| 0 (ref) | 1.00 | 1.00 |  |
| 1--2 | 1.80 (1.61, 2.02) | 1.37 (1.21, 1.55) |  |
| 3+ | 2.16 (1.74, 2.66) | 1.55 (1.24, 1.94) |  |
| ODSP record |  |  | <0.001 |
| No (ref) | 1.00 | 1.00 |  |
| Yes | 1.83 (1.642.05) | 1.57 (1.40, 1.77) |  |
| Renal Colic Care |  |  |  |
| # days in renal colic |  |  | <0.001 |
| 2 months (ref) | 1.00 | 1.00 |  |
| >2 months | 0.46 (0.44, 0.49) | 0.62 (0.59, 0.66) |  |
| Number of ED visits |  |  | <0.001 |
| 0 | 0.30 (0.27, 0.33) | 0.68 (0.61, 0.76) |  |
| 1 | 0.41 (0.37, 0.46) | 0.70 (0.62, 0.79) |  |
| 2 | 0.55 (0.48, 0.63) | 0.73 (0.64- 0.84) |  |
| >2 (ref) | 1.00 | 1.00 |  |
| Number of PCP visits |  |  | < 0.001 |
| 0 | 0.36 (0.33, 0.39) | 0.62 (0.56, 0.67) |  |
| 1 | 0.44 (0.41, 0.48) | 0.671 (0.62, 0.73) |  |
| 2 | 0.60 (0.55, 0.66) | 0.769 (0.70, 0.85) |  |
| > 2 (ref) | 1.00 | 1.00 |  |
| Subsequent surgery performed |  |  | < 0.001 |
| Yes | 2.30 (2.18, 2.43) | 1.86 (1.74, 1.98) |  |
| No (ref) | 1.00 | 1.00 |  |
| Total oral morphine equivalents |  |  | < 0.001 |
| 1 - <100 (ref) | 1.00 | 1.00 |  |
| 100 - <150 | 1.14 (1.03, 1.27) | 1.07 (0.96, 1.19) |  |
| 150 - <200 | 1.09 (0.98, 1.21) | 1.03 (0.93, 1.15) |  |
| 200 - <300 | 1.50 (1.36, 1.66) | 1.19 (1.06, 1.328) |  |
| 300 + | 3.12 (2.85, 3.41) | 1.59 (1.41, 1.79) |  |

PCP=Primary Care Physician

ED=Emergency Department

ODSP=Ontario Disability Support Program

CI=Confidence Intervals

^a^ Controlled for all covariates listed as well as enrollment in PCP practice, geographic region (urban, rural), specialty and gender of initial opioid prescriber as well as sum of opioid days supplied

^b^ *P* value for individual variable (not each categorical indicator) in the adjusted analyses (logistic regression)
