# Supplementary material for: The association of gender and persistent opioid use following an acute pain event: A retrospective population based study of renal colic
Source: PLoS One. 2021 Aug 26;16(8):e0256582. doi: 10.1371/journal.pone.0256582 (PMC8389463; doi:10.1371/journal.pone.0256582)
Supplement: S3 Table — (DOCX) [file pone.0256582.s004.docx]

**S3 Table. Odds Ratios for the Association Between Surgical Factors and Persistent Opioid Use by Gender**

|  | | | **Male** | | **Female** | | |
| --- | --- | --- | --- | --- | --- | --- | --- |
| **Surgical Characteristic** | **Value** | **Reference** | **Odds Ratio*** | **p-value^a^** | **Odds Ratio*** | | **p-value^a^** |
| Time to surgery from index visit | >= 30 days | < 30 days | 0.99 ( 0.86-1.14) | 0.89 | 0.936 (0.78-1.13) | | 0.48 |
| # of surgeries within 180 days of index renal colic | 2 | 1 | 1.28 (0.94-1.75) | < 0.001 | 1.89 (1.21-2.94) | | < 0.001 |
|  | > 2 |  | 3.18 (2.30- 4.38) |  | 5.56 (3.52-8.80) | |  |
| 1st surgery type | EWSL | URS | 1.74 (1.44-2.09) | < 0.001 | 1.52 (1.17- 1.97) | | 0.006 |
|  | PCNL |  | 1.01 (0.81- 1.26) |  | 0.99 (0.77- 1.28) | |  |
| Stent placement | Yes | No | 0.95 (0.69- 1.30) | 0.75 | 0.80 (0.52- 1.24) | | 0.323 |
| Septic stone | Yes | No | 1.10 (0.94- 1.28) | 0.22 | 0.94 (0.78- 1.13) | 0.52 | |

*Odds Ratio (95% Confidence Interval) for Persistent Opioid Use. Controlled for all covariates listed

^a^ P value for individual variable (not each categorical indicator) in the adjusted analyses (logistic regression)
