# Supplementary material for: The association of gender and persistent opioid use following an acute pain event: A retrospective population based study of renal colic
Source: PLoS One. 2021 Aug 26;16(8):e0256582. doi: 10.1371/journal.pone.0256582 (PMC8389463; doi:10.1371/journal.pone.0256582)
Supplement: Attachment — Submitted filename: PLOS rebuttal letter.aug3.docx [file pone.0256582.s006.docx]

May 26, 2021

Jingjing Qian

Academic Editor

PLOS ONE

Dear Dr. Qian:

Please find enclosed our manuscript re-submission (revision 2) entitled “The Association of Gender and Persistent Opioid Use Following an Acute Pain Event: A Population Based Study of Renal Colic”. Thank you for the opportunity to re-submit a revised version of this manuscript for consideration of publication in your journal.

Sincerely,

D. Robert Siemens

Professor and Chair

Department of Urology

Queen's University

Editors and Reviewers Comments:

Thanks for addressing majority of comments. Due to the small/modest adjusted difference between male and female found in the study, we would like the authors to take additional edits before we can accept your paper. Specifically:

1. Abstract:"Interpretation: After controlling for key covariates, females are at higher risk of demonstrating long term opioid use following an episode of renal colic. Evidence of prior mental health service utilization and acute colic care did not appear to significantly explain these observations." Please add "slightly" before "higher risk of demonstrating long term opioid use following an episode of renal colic" as you did in the conclusion section.

**This is very much appropriate and done (page 3)**

2. Methods: page 7, covariates -- the authors provided appropriate explanation in the responses to comments document, but did not incorporate corresponding edits in this section to justify selection of covariates. Please make the edits and cite relevant studies.

**Thank you. We have added a few sentences in the Methods explaining what we discussed in the rebuttal letter (with references).**

Journal Requirements:

**Further there was this one comment by a reviewer and we previously have addressed giving the ICES privacy policy. We are happy to add this as some supplementary comment or in “Acknowledgements”?**

4. Have the authors made all data underlying the findings in their manuscript fully available?

No

The PLOS Data policy requires authors to make all data underlying the findings described in their manuscript fully available without restriction, with rare exception (please refer to the Data Availability Statement in the manuscript PDF file). The data should be provided as part of the manuscript or its supporting information, or deposited to a public repository. For example, in addition to summary statistics, the data points behind means, medians and variance measures should be available. If there are restrictions on publicly sharing data—e.g. participant privacy or use of data from a third party—those must be specified.

***The dataset from this study is held securely in coded form at ICES. While legal data sharing agreements between ICES and data providers (e.g., healthcare organizations and government) prohibit ICES from making the dataset publicly available, access may be granted to those who meet pre-specified criteria for confidential access, available at www.ices.on.ca/DAS (email: das@ices.on.ca). The full dataset creation plan and underlying analytic code are available from the authors upon request, understanding that the computer programs may reply upon coding templates or macros that are unique to ICES and are therefore either inaccessible or may require modification.***
